# Supplementary material for: A data-informed mean-field approach to mapping of cortical parameter landscapes
Source: PLoS Comput Biol. 2021 Dec 23;17(12):e1009718. doi: 10.1371/journal.pcbi.1009718 (PMC8741023; doi:10.1371/journal.pcbi.1009718)
Supplement: S1 Text — (PDF) [file pcbi.1009718.s001.pdf]

Supplementary information: A data-informed mean-field approach to mapping of cortical parameter landscapes

Zhuo-Cheng Xiao Kevin K. Lin Lai-Sang Young

December 15, 2021

A Details of network model: network architecture and governing equations

In this section and the next, we describe the V1 network model we view as "ground truth". Except for minor simplifications, we follow [1]; interested readers are directed to [1] and references therein for more details.

"External" input to layer 4Cα neurons. We model a small piece of layer 4Cα of the Macaque primary visual cortex (V1), focusing on the background regime, i.e., spontaneous network activity. Layer 4Cα neurons deliver excitatory and inhibitory signals to each other. In addition, each Layer 4Cα neuron receives excitatory input from three categories of external (meaning external to 4Cα) sources, which we label *LGN*, *Layer 6*, and *ambient*. "LGN" refers to input from the lateral geniculate nucleus, which carries visual signals from the retina; "Layer 6" represents recurrent excitation from Layer 6 of V1; "ambient" is an amalgamation of weak cortical neuromodulatory signals. Since our primary goal is to investigate background firing rates (when external input rates are low), we model signals from all three sources as statistically independent, approximately Poisson (Bernoulli) processes, i.e., to generate a point process of rate R with timestep Δt, we place 0 or 1 spike in each time bin with probability $R\Delta t$.

Architecture of layer 4Cα network in V1. Consider a part of a 2D cortical sheet representing 3×3 hypercolumns, each occupying 0.5×0.5 mm^2 in layer 4Cα. This region contains 26,244 E-cells and 8,649 I-cells, which we assume are uniformly distributed, resulting in 3,877 cells (2,916 E, 961 I) per hypercolumn. In our model, E-cells are assumed to be spiny stellate cells and I-cells PV basket cells. The strength of connectivity between cells depends on the cell types (E or I, to be discussed later in *Equations* and Sect. B), while the probability of connection between any two cells in a local circuit is determined by a truncated Gaussian function of cell-to-cell distances. (In layer 4Cα there are no long-range connections.) For E-to-Q and I-to-Q connections, the standard deviations of the Gaussian functions are $0.2/\sqrt{2}$ mm and $0.125/\sqrt{2}$ mm, respectively, reflecting the different reach of E and I cells. The peak connection probability for E→E is 0.15, while the numbers for E→I, I→E, and I→I are set as 0.6, due to the much denser I-cell connections. We truncate all the Gaussian functions at $X^o = 0.36$ mm. Specifically, for a pair of neurons (i, j) which are x mm away from each other, the projection probability

from j to i is

$$P^{EE}(x) = 0.15 \times \exp\left\{-\left(\frac{x}{0.2}\right)^2\right\},$$
$$P^{EI}(x) = 0.60 \times \exp\left\{-\left(\frac{x}{0.125}\right)^2\right\},$$
$$P^{IE}(x) = 0.60 \times \exp\left\{-\left(\frac{x}{0.2}\right)^2\right\},$$
$$P^{II}(x) = 0.60 \times \exp\left\{-\left(\frac{x}{0.125}\right)^2\right\},$$

and

$$P^{ij}(x) = 0 \quad \text{for } x > X^o;\ i,j \in \{E,I\}.$$

The connection probabilities and cell densities result in that, on average, each E-cell has ∼210 presynaptic E-cells and ∼110 I presynaptic cells, while each I -cell has ∼840 presynaptic E-cells and ∼110 presynaptic I-cells. We leave the neurons close to the boundary as is and choose not to compensate for the missed presynaptic neurons.

Equations. We use conductance-based leaky-integrate-and-fire (LIF) models for neuronal dynamics within the Layer 4Cα network. For a neuron i with cell type $Q \in \{E, I\}$, its membrane potential v_i advances by

$$\frac{\mathrm{d}v_i}{\mathrm{d}t} = -g_Q^L(v_i - V_{\text{rest}}) - g_i^E(t)(v_i - V^E) - g_i^I(t)(v_i - V^I)\ . \tag{A}$$

We nondimensionalize voltages, setting resting potential to $V_{\text{rest}} = 0$ and spiking threshold $V_{\text{th}} = 1$. Every time v_i reaches V_{th}, a spiking event occurs at neuron i and the signal is sent to all its postsynaptic neurons. Afterwards, v_i enters a refractory period for $\tau_{\text{ref}} = 2$ ms right away, then reset to V_{rest}.

With the selections of the reversal potentials $V^E = 14/3$ and $V^I = -2/3$ [2], the membrane potential v_i is driven by three current terms in Eq (A):

(i) Towards $V_{\text{rest}} = 0$ due to the leaky current $-g_Q^L(v_i - V_{\text{rest}})$, where g_Q^L stands for membrane leakage conductance of cell type Q.

(ii) Towards $V^I = -2/3$ due to the inhibitory current $-g_i^I(t)(v_i - V^I)$, where the inhibitory conductance $g_i^I(t)$ is determined by the spiking series generated by inhibitory cells presynaptic to neuron i, i.e.,

$$g_i^I(t) = S^{QI} \sum_{j \in N_{4C,I}(i)} \sum_{k=1}^{\infty} G_{\text{gaba}}(t - t^j(k))\ . \tag{B}$$

Here, S^{QI} stands for the connectivity from an I-cell to a type-Q cell. Cell j belongs to $N_{4C,I}(i)$, the collection of all presynaptic I-cells to neuron i, generating a spiking time series $\{t^j\}$. In addition, $G_{\text{gaba}}(t)$ is a Green's function modeling the temporal increment of inhibitory conductances induced by each I-spike through GABA receptors (details provided in Sect. B).

(iii) Towards $V^E = 14/3$ due to the excitation current $-g_i^E(t)(v_i - V^E)$. The excitatory conductance of neuron i, $g_i^E(t)$, consists of four components:

$$
\begin{aligned}
g_i^E(t) = &\underbrace{S^{\text{Qlgn}} \sum_{k=1}^{\infty} G_{\text{ampa}}(t - t^{i,\text{lgn}}(k))}_{\text{(I) LGN}} + \underbrace{S^{\text{Qamb}} \sum_{k=1}^{\infty} G_{\text{ampa}}(t - t^{i,\text{amb}}(k))}_{\text{(II) ambient}} \\
&\underbrace{+ S^{\text{QL6}} \sum_{k=1}^{\infty} \Big[\rho^Q_{\text{ampa}} G_{\text{ampa}}(t - t^{i,\text{L6}}(k)) + \rho^Q_{\text{nmda}} G_{\text{nmda}}(t - t^{i,\text{L6}}(k)) \Big]}_{\text{(III) Layer 6}} \\
&\underbrace{+ S^{QE} \sum_{j \in N_{4\text{C},E}(i)} \sum_{k=1}^{\infty} \Big[\rho^Q_{\text{ampa}} G_{\text{ampa}}(t - t^j(k)) + \rho^Q_{\text{nmda}} G_{\text{nmda}}(t - t^j(k)) \Big]}_{\text{(IV) Layer 4}} .
\end{aligned}
\tag{C}
$$

Terms I-IV represent synaptic conductances induced by LGN, ambient, Layer 6 input, and Layer 4 recurrent excitation, respectively. For each neuron i, the spiking series in terms I-III are modeled by Poisson processes as described above. For E-spikes from Layer 4 and 6, two different types of excitatory synapses (AMPA and NMDA) induce different temporal increment of $g_i^E(t)$ (term III and IV), while only AMPA synapse is involved for LGN and ambient input (term I and II). Here, $\rho^Q_{\text{amda,nmda}}$ stand for the fractions of synaptic input received by AMPA and NMDA receptors in a type-Q neuron; (S^{Qlgn}, S^{Qamb}, and S^{QL6}, S^{QE}) denote the respective synaptic coupling weights of these sources towards type-Q cells.

For the E-to-E input in Layer 4, two additional biological details are incorporated in the model. First, we consider a possibility for synaptic failure $p_{\text{fail}} = 20\%$, i.e., whether the k-th spike from neuron j successfully induces a change in $g_i^E(t)$ depends on an independent coin-toss with $p = 0.8$. Second, if a spike is "successful", a random delay is added to $t^j(k)$ to model the fact that E-neurons project to the dendrites of other E-cells, instead of the soma. In all, when neuron i is an E-neuron, the term IV in Eq (C) is replaced by

$$
S^{EE} \sum_{j \in N_{4\text{C},E}(i)} \sum_{k=1}^{\infty} \sigma^j(k) \Big[\rho^E_{\text{ampa}} G_{\text{ampa}}(t - (t^j(k) + \tau^j(k))) + \rho^E_{\text{nmda}} G_{\text{nmda}}(t - (t^j(k) + \tau^j(k))) \Big],
$$

where $\sigma^j(k)$ stands for the coin-toss, and the $\tau^j(k)$ are independent, identically distributed random delay times uniformly distributed on $[0, 1]$ ms.

B Parameters

We now list the specific parameter values used. We remark that while EPSC and IPSCs have been measured in the laboratory and so can be assumed to be known, the coupling weights — which involve how one neuron affects another — cannot be measured directly. This is the main reason we mostly regard the coupling weights as free parameters to be investigated in this paper, in spite of experimental evidence that may provide certain ranges for them.

Neuronal parameters. The following parameters are used for L4 neurons, and are fixed throughout the paper.

(i) Reversal potentials: $V^I = -2/3$, $V^E = 14/3$

(ii) Leakage conductances: $g_E^L = (20 \text{ ms})^{-1}$, $g_I^L = (16.7 \text{ ms})^{-1}$

(iii) Postsynaptic conductances:

$$G_s(t) = \frac{1}{\tau_s^{\text{decay}} - \tau_s^{\text{rise}}} \left(e^{-\frac{t}{\tau_s^{\text{rise}}}} - e^{-\frac{t}{\tau_s^{\text{decay}}}} \right),$$

where $(\tau_s^{\text{rise}}, \tau_s^{\text{decay}})$ stand for the time scales of activation/inactivation of synapse type $s = \text{ampa}, \text{nmda}, \text{gaba}$; the time constants used here are

- $(\tau_{\text{ampa}}^{\text{rise}}, \tau_{\text{ampa}}^{\text{decay}}) = (0.5, 3)$ ms
- $(\tau_{\text{nmda}}^{\text{rise}}, \tau_{\text{nmda}}^{\text{decay}}) = (2, 80)$ ms
- $(\tau_{\text{gaba}}^{\text{rise}}, \tau_{\text{gaba}}^{\text{decay}}) = (0.5, 5)$ ms

(iv) Fraction of AMPA and NMDA receptors activated by E-spikes: $(\rho_{\text{ampa}}^E, \rho_{\text{nmda}}^E) = (0.8, 0.2)$, and $(\rho_{\text{ampa}}^E, \rho_{\text{nmda}}^E) = (0.67, 0.33)$

(v) Synaptic failure: $\sigma^j(k) = 1$ with $p = 0.8$, and $\sigma^j(k) = 0$ with $p = 0.2$

(vi) Synaptic delays: the $\tau^j(k)$ are uniformly distributed between $[0, 1]$ ms

(vii) Refractory period: $\tau_{\text{ref}} = 2$ ms

For biophysical constants, see, e.g., [2]. We follow [1, 3] for all other parameters.

Network parameters. These include all synaptic coupling weights and rates of external input, making up the parameter space in which we compute the landscape of E- and I-firing rates. We specify below our choices of them. Those parameters that are specified with ranges form the 7D space of *free parameters* we investigate in this paper. These *a priori* constraints of free parameters are discussed later in this section. Generally, we first choose the values of S^{EE} and S^{II} (independently of other parameters), then index some of the other parameters to S^{EE} and S^{II}.

(i) Synaptic coupling weights chosen independently: $S^{EE} \in (0.018,\ 0.030)$, $S^{II} \in (0.08,\ 0.20)$

(ii) Synaptic coupling weights depending on S^{EE}:

- $S^{E\text{lgn}} \in (1.5,\ 3) \times S^{EE}$, $S^{I\text{lgn}} \in (1.5,\ 3) \times S^{E\text{lgn}}$
- $S^{E\text{L6}} = \frac{1}{3} \times S^{EE}$
- $S^{EI} \in (0.9,\ 2.4) \times S^{EE}$

(iii) Synaptic coupling weights depending on S^{II}:

- $S^{IE} \in (0.1,\ 0.25) \times S^{II}$

- $S^{I\mathrm{L6}} = \frac{1}{3} \times S^{IE}$

(iv) LGN input rates: $F^{E\mathrm{lgn}} = F^{I\mathrm{lgn}} = 80$ Hz

(v) Layer 6 input rates: $F^{E\mathrm{L6}} = 250$ Hz, $F^{I\mathrm{L6}} \in (1.5,\ 6) \times F^{E\mathrm{L6}}$

(vi) Ambient input:

- $F^{E\mathrm{amb}} = F^{I\mathrm{amb}} = 500$ Hz
- $S^{E\mathrm{amb}} = S^{I\mathrm{amb}} = 0.01$

Prior biological constraints and scaling conventions. We justify our choices of the ranges of *free parameters* above. Following (often indirect) suggestions from experimental observations and heuristic reasoning, one can arrive at some bounds on them. The ranges we impose are broader than those suggested by available data; the greater the uncertainty, the wider the net we cast. Specifically:

- $S^{EE} \in (0.015, 0.03)$: This follows from the conventional wisdom that when an E-cell is stimulated *in vitro*, it takes 10-50 consecutive spikes in relatively quick succession to produce a spike. Numerical simulation suggests the assumed order of magnitude for S^{EE} is reasonable [4].
- $S^{EI} \in (0.9,\ 2.4) \times S^{EE}$ and $S^{IE} \in (0.1,\ 0.25) \times S^{II}$: In the absence of experimental guidance, we located these ranges numerically as follows: We examined firing rate maps for wider ranges than these, and found that, for the most part, the geometry on inhibition planes forces the good areas to lie within these ranges.
- $S^{II} \in (0.08,\ 0.20)$: There is no direct empirical information on this parameter; however, there is evidence that EPSPs for I-cells are similar in size to those for E-cells [5]. We choose the range for S^{II} by following the logistic that $S^{II} \in (0.08,\ 0.20)$ and $S^{IE} \in (0.1,\ 0.25) \times S^{II}$ means $S^{IE} \in (0.008,\ 0.05)$, which contains and is significantly larger than the range of S^{EE} above.
- $S^{E\mathrm{lgn}} \in (1.5, 3.0) \times S^{EE}$: Results from [6] suggest that the sizes of EPSPs from LGN are $\sim 2\times$ those from L4. We therefore assume a range around 2.
- $S^{I\mathrm{lgn}} \in (1.5, 3.0) \times S^{E\mathrm{lgn}}$: we assume $S^{I\mathrm{lgn}} > S^{E\mathrm{lgn}}$ because it has been reported that LGN produces larger EPSCs in I-cells [7].
- $F^{I\mathrm{L6}} \in (1.5, 6) \times F^{E\mathrm{L6}}$: Within L4, an I-cell has 3.5-4 times as many presynaptic E-neurons as an E-cell. If we hypothesize a similar ratio between L6 and L4, it would follow that $F^{I\mathrm{L6}} \in (3.5, 4) \times F^{E\mathrm{L6}}$. We relax the interval to $(1.5, 6)$ because of uncertainty surrounding L6: whether the effect of L6 on L4 is net-excitatory or net-inhibitory is an issue that is currently unresolved for the real cortex. A wider range also serves to absorb potential errors in the assumption that $S^{I\mathrm{L6}} = \frac{1}{3} \times S^{IE}$.

C Closer look at MF+v: solvability of MF equation and implementation details

Biologically meaningful solutions of MF equation. In some situations, the MF equation (4) yields negative firing rates when given valid mean voltages; we have indicated such parameters

by gray in all parameter plots. Here, we discuss some of the reasons underlying these failures.

First, recall that in Methods, we had asserted that the MF equation Eq (8) can be written in matrix form as

$$\vec{\mathbf{f}} = \mathbf{R}(\vec{\mathbf{f}}) \times \left[\mathbf{M}(\vec{\mathbf{v}}) \cdot \vec{\mathbf{f}} + \vec{\mathbf{s}}(\vec{\mathbf{v}})\right].$$

(This was Eq (9) in Methods.) This can be seen by defining

$$\begin{aligned}
\mathbf{R}(\vec{\mathbf{f}}) &= \begin{bmatrix} 1 - f_E \tau_{\text{ref}} & 0 \\ 0 & 1 - f_I \tau_{\text{ref}} \end{bmatrix} \\
\mathbf{M}(\vec{\mathbf{v}}) &= \begin{bmatrix} S^{EE} N^{EE} (1 - p_{\text{fail}})(V^E - \overline{v}_E) & S^{EI} N^{EI} (V^I - \overline{v}_E) \\ S^{IE} N^{IE} (V^E - \overline{v}_I) & S^{II} N^{II} (V^I - \overline{v}_I) \end{bmatrix} \\
\vec{\mathbf{s}}(\vec{\mathbf{v}}) &= \begin{bmatrix} \left(S^{E\text{lgn}} F^{E\text{lgn}} + S^{E\text{L6}} F^{E\text{L6}} + S^{E\text{amb}} F^{E\text{amb}}\right)(V^E - \overline{v}_E) - g_E^L \overline{v}_E \\ \left(S^{I\text{lgn}} F^{I\text{lgn}} + S^{I\text{L6}} F^{I\text{L6}} + S^{I\text{amb}} F^{I\text{amb}}\right)(V^E - \overline{v}_I) - g_I^L \overline{v}_I \end{bmatrix}
\end{aligned} \tag{D}$$

and verifying directly. Our interest is in finding nonnegative solutions $\vec{\mathbf{f}}$ of this equation given mean voltages $\vec{\mathbf{v}}$. The solvability of Eq (9) depends on the properties of the matrix $\mathbf{M}(\vec{\mathbf{v}})$, which in turn depends on L4 connectivity and the mean voltages $\vec{\mathbf{v}}$. Note that the entries of $\vec{\mathbf{s}}(\vec{\mathbf{v}})$ represent the mean currents into E and I cells, respectively, and must be positive for cells to fire with positive rates.

Among the scenarios in which these equations fail to give meaningful firing rates, by far the simplest is when the equations are (nearly) singular. Ignoring the refractory factor $\mathbf{R}(\vec{\mathbf{f}})$ (whose effect is perturbative), Eq (9) is equivalent to

$$\vec{\mathbf{f}} = [I - \mathbf{M}(\vec{\mathbf{v}})]^{-1} \times \vec{\mathbf{s}}(\vec{\mathbf{v}}) \,, \tag{E}$$

where

$$[I - \mathbf{M}(\vec{\mathbf{v}})] = \begin{bmatrix} 1 - [4\text{E} \to \text{E}] & -[4\text{I} \to \text{E}] \\ -[4\text{E} \to \text{I}] & 1 - [4\text{I} \to \text{I}] \end{bmatrix} = \left[\ \vec{\alpha}_1 \mid \vec{\alpha}_2\ \right] ,$$

provided $I - \mathbf{M}(\vec{\mathbf{v}})$ is nonsingular. In the above, $[\text{P} \to \text{Q}]$ indicate the corresponding entry of matrix $M(\vec{\mathbf{v}})$, i.e., the net contribution to an E/I-cell from one E/I-kick. When $\det(I - \mathbf{M}(\vec{\mathbf{v}})) = 0$, the linearized equation above may not have a solution, suggesting MF+v iteration is likely to fail when $I - \mathbf{M}(\vec{\mathbf{v}})$ is (nearly) singular.

One can in fact take a more geometric view of the solvability of Eq (9), one that makes questions surrounding solvability more transparent. Let $\vec{\alpha}_1$ and $\vec{\alpha}_2$ be the columns of $I - \vec{\mathbf{M}}(\vec{\mathbf{v}})$. Eq (9) yields nonnegative firing rates precisely when there exist $f_1, f_2 \geqslant 0$ such that $f_1\vec{\alpha}_1 + f_2\vec{\alpha}_2 = \mathbf{s}(\vec{\mathbf{v}})$. This can be visualized by defining $S = \{f_1\vec{\alpha}_1 + f_2\vec{\alpha}_2 \mid f_1, f_2 \geqslant 0\}$ and noting that the intersection of S with the first quadrant $\{s_1, s_2 \geqslant 0\}$ is precisely the set of all $\vec{\mathbf{s}}$ such that $\vec{\mathbf{f}} = (I - \vec{\mathbf{M}}(\vec{\mathbf{v}}))^{-1}\vec{\mathbf{s}}$ results in nonnegative firing rates. Fig A(i) shows an example. Here, the two (normalized) column vectors $\vec{\alpha}_1$ and $\vec{\alpha}_2$ are linearly independent, and the set S (the region bounded by span($\vec{\alpha}_1$) and span($\vec{\alpha}_2$) and contains the black line) has a large intersection with the first quadrant, so that *most* nonnegative values of $\vec{\mathbf{s}}$ lead to nonnegative rates. Note that these parameters lie well above the good area; *cf.* Figs 1 and A.

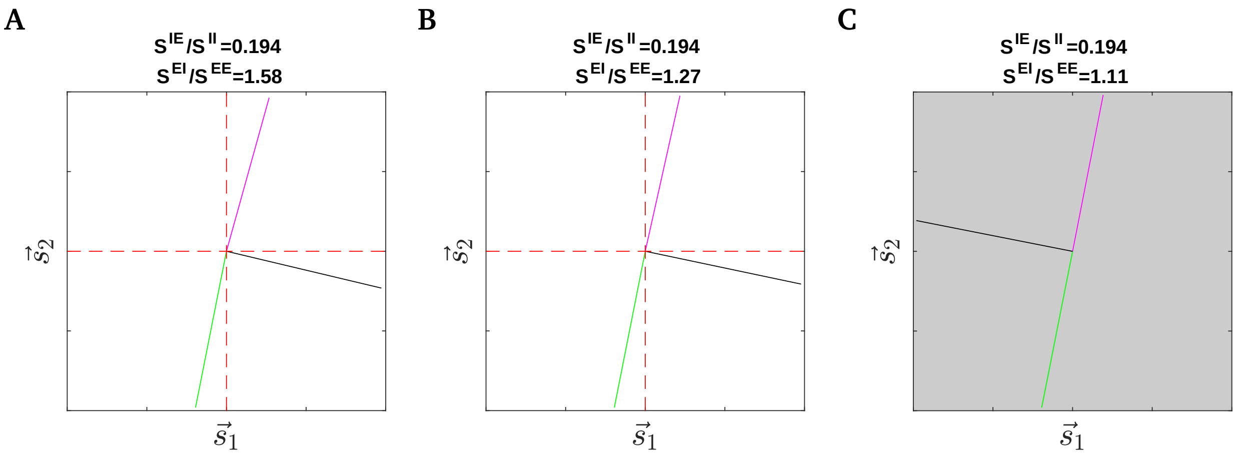

Figure A: A geometric view of the solvability of MF equations. Each panel contains two normalized column vectors of $I - \mathbf{M}(\vec{\mathbf{v}})$, i.e., $\vec{\alpha}_1$ (green vector in the third quadrant) and $\vec{\alpha}_2$ (purple vector in the first quadrant). The black line segment marks the region $S = \{f_1\vec{\alpha}_1 + f_2\vec{\alpha}_2 \mid f_1, f_2 \geqslant 0\}$. **(i)** Eq (9) is nonsingular and well-behaved. **(ii)** Eq (9) is near-singular and its column vectors are nearly collinear. **C.** $\det(I - \mathbf{M}(\vec{\mathbf{v}}))$ changes sign and MF+v gives negative firing rates.

To see what else might happen, we now move along a line in the inhibition plane defined by $S^{IE}/S^{II} = 0.194$, starting from the value $S^{EI}/S^{EE} = 1.58$ used in Fig A(i) and moving down. Fig A(ii) shows what happens for $S^{EI}/S^{EE} = 1.27$, which lies within the good area: $\vec{\alpha}_1$ and $\vec{\alpha}_2$ become more nearly collinear, though there is still a sizable intersection between S and the first quadrant, so that most values of $\vec{\mathbf{s}}$ lead to positive firing rates. However, as S^{EI} decreases even further, $\det(I - \mathbf{M}(\vec{\mathbf{v}}))$ changes sign, and the set S abruptly flips to the other side of the dividing line, leading Eq (9) to produce negative firing rates for many values of $\vec{\mathbf{s}}$.

In Fig B, we extend this picture to the inhibition plane. For each choice of S^{EI}/S^{EE} and S^{IE}/S^{II}, we compute $\mathbf{M}(\vec{\mathbf{v}})$ by assuming $\vec{\mathbf{v}} = (\nu_E, \nu_I) = (0.55, 0.65)$. Each panel shows the two (normalized) column vectors $\vec{\alpha}_1$ and $\vec{\alpha}_2$, along with a black line marking the region $S(\vec{\alpha}_1, \vec{\alpha}_2)$. Observe that as S^{EI}/S^{EE} and S^{IE}/S^{II} decrease, $\vec{\alpha}_1$ and $\vec{\alpha}_2$ first become linearly dependent (so that $I - \mathbf{M}(\vec{\mathbf{v}})$ becomes singular), then changing orientation and resulting in negative firing rate estimates (gray panels).

Two final remarks. First, the boundary between gray and white panels corresponds roughly to where $\det(I - \mathbf{M}(\vec{\mathbf{v}}))$ changes sign. This is also where explosive dynamics occur in our *network* simulations due to low suppression index SI_E or very low E-firing rates (caused by high external input to I-cells; more below). This suggests that parameters where the MF+v algorithm fails to give biologically meaningful estimates are also parameters where the network model itself fails to give biologically meaningful results.

Second, concerning the narrow wedge of $\vec{\mathbf{s}}$-values in the first quadrant that yield negative firing rate estimates, i.e., the set bounded by $\vec{\alpha}_2$ (oblique purple vector in the first quadrant) and the vertical axis in Fig A(i,ii). The same pattern also occur in the upper part of Fig B: These correspond to when the external inputs to I-cells are too high. Though the corresponding sets of $\vec{\mathbf{s}}$-values are small, they can have a significant impact. For example, for high-$S^{I\mathrm{lgn}}$ and/or

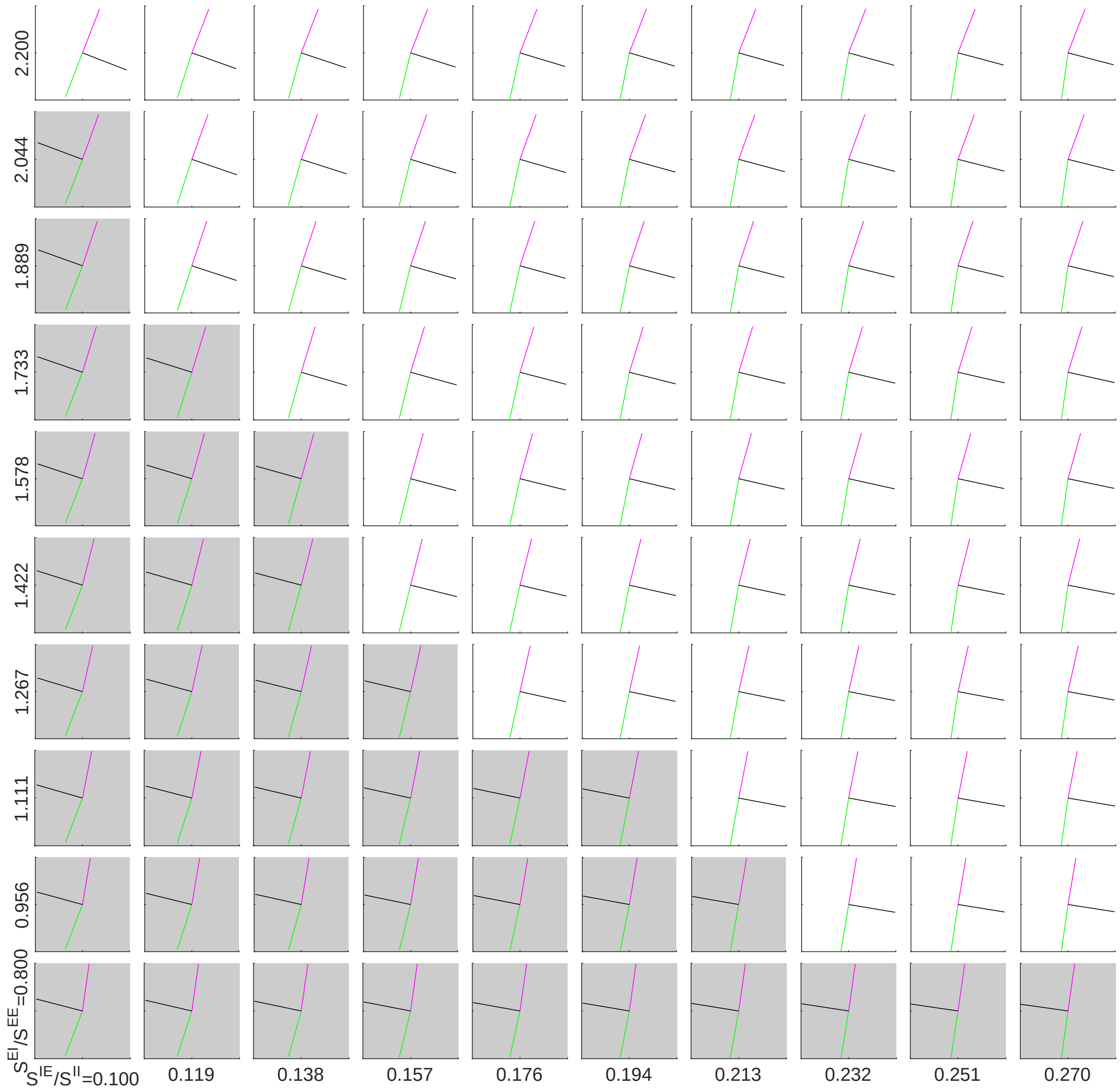

Figure B: Solvability of Eq (9) on the inhibition plane. Here, $S^{EE} = 0.024$ and $S^{II} = 0.120$, and we fix $\vec{\mathbf{v}} = (\nu_E, \nu_I) = (0.55, 0.65)$ for all panels. Panels with gray color corresponds to negative firing rates from the MF equations, and the boundary between gray and white panels roughly corresponds to where $\det(I - \mathbf{M}(\vec{\mathbf{v}}))$ changes sign.

high-F^{IL6} regimes, this can lead to large swaths of gray. See Figs 2 and 3, and Sect. E.

Implementation and design of MF+v. As explained in Methods (see M2), our first attempt at a fixed point iteration did not result in a convergent algorithm. So we iterate until the firing rate estimates stabilize to a narrow, nearly linear band in firing rate space, then average a number of successive estimates to produce an estimate.

Algorithm 1: The MF+v method.

Result: MF computed firing rates $\vec{\mathbf{f}}$, and mean voltages $\vec{\mathbf{v}}$.

Set parameters S^{EE}, $S^{I\mathrm{lgn}}$, F^{EL6}, etc.;

$(\vec{\mathbf{f}}_0, \vec{\mathbf{v}}_0) \leftarrow$ initial conditions;

$M \leftarrow$ maximum number of training iterations; `// see text for value`

$t^{\mathrm{LIF}} \leftarrow$ integration time; `// default` $t^{\mathrm{LIF}} = 20\mathrm{s}$

$\varepsilon \leftarrow$ early termination tolerance; `// default` $\varepsilon = 0.05$

$k \leftarrow$ early termination horizon; `// default` $k = 15$

`// Training loop`

for $p \leftarrow 1$ **to** M **do**

    $\vec{\mathbf{v}}_p = \mathrm{LIF}(\vec{\mathbf{f}}_{p-1}, t^{LIF})$;

    $\vec{\mathbf{f}}_p = \mathrm{MF}(\vec{\mathbf{v}}_p, \vec{\mathbf{f}}_{p-1})$;

    if $CV(\vec{\mathbf{f}}_p, \cdots, \vec{\mathbf{f}}_{p-k}) < \varepsilon$ **then**

        goto `FINALIZE`;

end

`FINALIZE:`

if $p < M$ **then**

    `// Convergence criterion satisfied`

    `// Compute a more accurate estimate via a longer time average`

    $\ell_1 \leftarrow$ number of voltages to use in moving average; `// default` $\ell_1 = 10$

    $\ell_2 \leftarrow$ number of firing rates to use in final estimate; `// default` $\ell_2 = 50$

    for $q \leftarrow p+1$ **to** $p+\ell_2$ **do**

        $\vec{\mathbf{v}}_q = \mathrm{LIF}(\vec{\mathbf{f}}^*_{q-1}, t^{\mathrm{LIF}})$;

        $\vec{\mathbf{v}}^*_q = \mathrm{mean}(\vec{\mathbf{v}}_{q-\ell_1}, \cdots, \vec{\mathbf{v}}_q)$; `// moving average`

        $\vec{\mathbf{f}}^*_q = \mathrm{MF}(\vec{\mathbf{v}}^*_q, \vec{\mathbf{f}}^*_{q-1})$;

    end

    return $\mathrm{mean}(\vec{\mathbf{f}}^*_{p+1}, \cdots, \vec{\mathbf{f}}^*_{p+\ell_2})$;

else

    `// Unconverged`

    return `FAIL`

Algorithm 1 gives a precise summary and lists all other hyperparameter values used. A practical issue is that we need to check the variance of the voltage and firing estimates to determine when to stop iterating. To make this efficient, instead of carrying out accurate but expensive long-time average for every iteration, we use shorter runs that may be noisy by themselves but can be averaged together to produce accurate estimates. We then use a small

number of consecutive iterations to check convergence during an initial, “training” phase, and when certain stopping criteria are satisfied, we compute a more accurate estimate and output the result.

In this paper, the precise stopping criterion is based on comparing the *coefficient of variation*

$$CV(\vec{\mathbf{v}}_p, \cdots, \vec{\mathbf{v}}_{p-k}) = \frac{\mathrm{var}(\vec{\mathbf{v}}_p, \cdots, \vec{\mathbf{v}}_{p-k})^{1/2}}{\mathrm{mean}(\vec{\mathbf{v}}_p, \cdots, \vec{\mathbf{v}}_{p-k})} \tag{F}$$

of the last k voltage estimates is against a pre-specified tolerance ε. When the stopping criterion is satisfied, we use moving averages of the voltages to compute a larger number of iterations, and use these to estimate the firing rate. For the network models studied in this paper, we have found the estimates to be insensitive to the exact choice of the maximum iteration number M. We typically set M in the range 300–500.

D Miscellaneous information on MF+v

Here we record some additional information that have affected our decision to use MF+v in this paper.

Effects of refractory period and different kick sizes. The mean voltages $\vec{\mathbf{v}}$ produced by the LIF equations (and hence the MF-computed firing rates $\vec{\mathbf{f}}$) can depend on parameters in a nontrivial way, making it difficult to estimate $\vec{\mathbf{v}}$ using analytical methods. Here, we illustrate the parameter dependence of LIF neurons via two examples, using the parameters in Sect. B. In both examples, a pair of LIF neurons (one E and one I) are each presented with Poissonian spike trains modeling L4 inputs, with input rates f_E^{in} and f_I^{in}, in addition to L6, LGN, and amb inputs. The two neurons are uncoupled and given independent inputs. We denote the resulting output rates f_E^{out} and f_I^{out}.

Our first example concerns the refractory period τ_{ref}, which can significantly impact neuronal activity because membrane conductances and currents steadily decay during refractory periods; the larger the τ_{ref}, the more conductance is “missed” by the neuron while refractory. For instance, for an E-cell with a 3 Hz firing rate, its membrane potential stays unchanged for $3 \times \tau_{\mathrm{ref}}$ in each second. This can be a non-negligible fraction of time, and the effect is exacerbated by higher firing rates. Fig C(i) shows the mean voltages and firing rates as τ_{ref} varies from 0 to 4 ms. Though f_E^{out} does not change much (5.5-5.8 Hz), f_I^{out} experiences a sharp change (19-25 Hz).

Our second example is motivated by the observation that for neurons in a mean-driven regime (a limit often studied in theoretical analyses of neuron models), their output rate depends on L4 kick sizes $S^{QQ'}$ ($Q, Q' \in \{E, I\}$) only through the product $S^{QQ'} \times f_{Q'}^{in}$. Thus, if we vary $S^{QQ'}$ while keeping $S^{QQ'} \times f_{Q'}^{in}$ constant, any variation in output rates ($\vec{\mathbf{f}}^{out}$) would be due to fluctuations in the L4 inputs. To test this, we perform the scaling $S^{QQ'} \mapsto \alpha S^{QQ'}$ and $f_{Q'}^{in} \mapsto f_{Q'}^{in}/\alpha$ for a range of scaling factors α. Other parameters, including ($S^{\mathrm{Qlgn}}, F^{\mathrm{Qlgn}}$) and ($S^{\mathrm{QL6}}, F^{\mathrm{Qlgn}}$) (which were previously indexed to S^{QE}) are kept constant. Fig C(ii) shows the results. Observe that both $\vec{\mathbf{v}}$ and $\vec{\mathbf{f}}^{out}$ experience sharp changes when α moves away from 1. In particular, the firing rates are almost 0 when α is small (low fluctuation), and unreasonably high when α is large (high fluctuation). These results suggest that for the background regime studied here, MF+v (as is the network that it models) operates in a fluctuation driven regime.

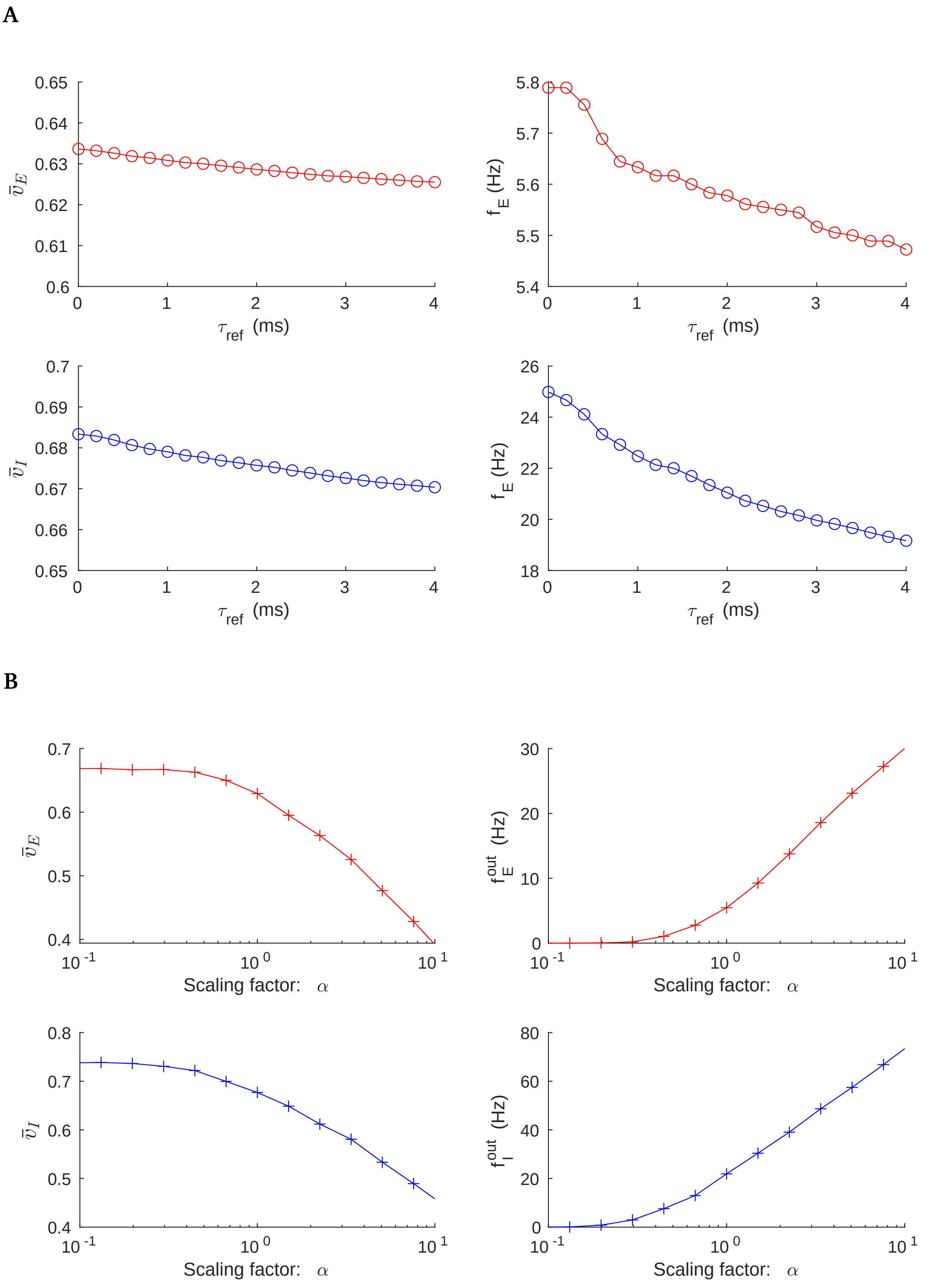

Figure C: Mean voltages and firing rates computed by MF+v with different **(i)** refractory periods (τ_{ref}) and **(ii)** L4 excitatory kick sizes (S^{EE} scaling factor α). Here, $S^{EE} \times f_E^{in} = 0.13$, $S^{EI} \times f_I^{in} = 0.65$, $S^{IE} \times f_E^{in} = 0.10$, and $S^{II} \times f_I^{in} = 2.25$.

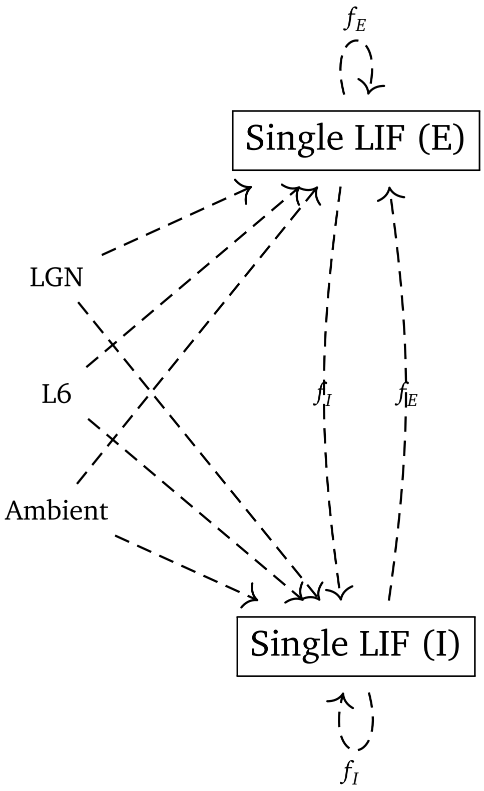

Figure D: The LIF-only algorithm. We begin by choosing initial values f_E^0 and f_I^0. In the first iteration, these values are used to drive a pair of LIF neurons for 20 seconds (biological time). The resulting firing rates (instead of the membrane voltages in MF+v) are then fed into the next iteration as the L4 input. In the above, all dashed lines are modeled by Poisson processes.

Algorithm 2: The LIF-only algorithm. We have removed the early termination criterion to simplify comparison with MF+v.

Result: LIF-computed $\vec{\mathbf{f}}$.

Set parameters S^{EE}, $S^{I\text{lgn}}$, $F^{E\text{L6}}$, etc.;

$\vec{\mathbf{f}}_0 \leftarrow$ initial conditions;

$M \leftarrow$ maximum number of training iterations; `// default` $M = 100$

$t^{\text{LIF}} \leftarrow$ integration time; `// default` $t^{\text{LIF}} = 20\text{s}$

`// Training loop`

for $p \leftarrow 1$ **to** M **do**

  $\vec{\mathbf{f}}_p = \text{LIF}(\vec{\mathbf{f}}_{p-1}, t^{LIF})$;

end

$\ell_1 \leftarrow$ number of voltages to use in moving average; `// default` $\ell_1 = 10$

$\ell_2 \leftarrow$ number of firing rates to use in final estimate; `// default` $\ell_2 = 50$

for $q \leftarrow p+1$ **to** $p+\ell_2$ **do**

  $\vec{\mathbf{f}}_q = \text{LIF}(\vec{\mathbf{f}}^*_{q-1}, t^{\text{LIF}})$;

  $\vec{\mathbf{f}}^*_q = \text{mean}(\vec{\mathbf{f}}_{q-\ell_1}, \cdots, \vec{\mathbf{f}}_q)$; `// moving average`

end

return $\text{mean}(\vec{\mathbf{f}}^*_{p+1}, \cdots, \vec{\mathbf{f}}^*_{p+\ell_2})$;

Comparison to LIF-only. A natural alternative to MF+v is an LIF-only method: We drive a pair of LIF models (one E and one I) with Poissonian spike trains of rate $\vec{\mathbf{f}}$ as L4 input, along with Poisson spike trains modeling inputs from LGN, L6, and amb, and look for values of $\vec{\mathbf{f}}$ that lead to output rates equal to $\vec{\mathbf{f}}$. That is, we look for a self-consistent MF approximation without reference to Eq (4). This can be implemented by simply iterating LIF neurons and feed $\overline{f}_p$ to iteration $p+1$ directly as the L4 E/I input. A schematic representation is in Fig D, with details in Algorithm 2.

We find that all else being equal, the LIF-only algorithm is much less stable than MF+v. To demonstrate this, we select two parameters from Fig 9C ($S^{EI} \in \{0.0433, 0.0402\}$) and compare a simplified version of MF+v and LIF-only. In these runs, to avoid uncertainties associated with early termination, we train both algorithms for $M = 100$ iterations, then compute running averages. For MF+v, this means that in Algorithm 1, we set $M = 100$ and $\varepsilon = 0$, and always take the first branch after `FINALIZE`; see Algorithm 2 for LIF-only. Fig E shows the results. In the left panels, we plot the firing rates $\vec{\mathbf{f}}_p$ for iterates $p \leqslant 100$ and running averages $\vec{\mathbf{f}}^*_p$ for $100 < p \leqslant 400$. As can be seen, firing rates from MF+v stabilizes quickly to network-computed rates, while LIF-only (right) sometimes exhibits large oscillations.

A potential explanation for the behavior of LIF-only is that (as we noted in Methods Sect. M2) one can obtain many more samples of voltages per unit time than spikes. Since the variance of firing rate estimates is roughly inversely proportional to the number of spikes, the single neuron rate estimates in the LIF-only algorithm are far noisier at typical background firing rates. We have also tested other variants of LIF-only, such as averaging over ensembles of pairs of LIF neurons. However, the LIF-only method remains rather unstable (data not shown). Although LIF-only occasionally gives good predictions of firing rates, it is far less reliable in comparison

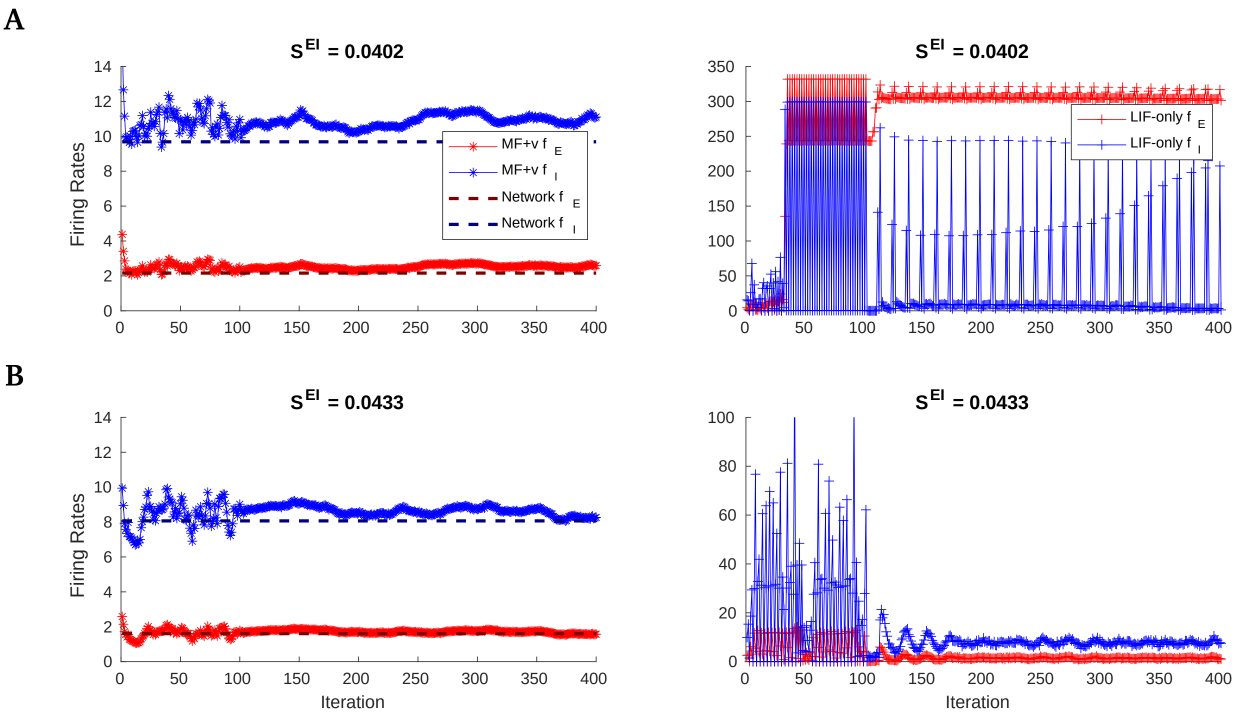

Figure E: Comparison of MF+v and LIF-only. Both are trained for 100 iterations with identical LIF neuron simulation time $t^{\text{LIF}} = 20\text{s}$. **(i)** E/I firing rate trajectories from $\vec{\mathbf{f}}_p$ (iteration 0-100) and $\vec{\mathbf{f}}_p^*$ (iteration 101-400), for $S^{EI} = 0.0402$. Left: MF+v exhibits stable estimates for both parameter choices. Right: LIF-only exhibit large oscillations. **(ii)** Same as **A**, but for $S^{EI} = 0.0433$.

to MF+v.

E Additional firing rate maps

As mentioned in Results, here we show versions of Fig 2 with different choices of parameters.

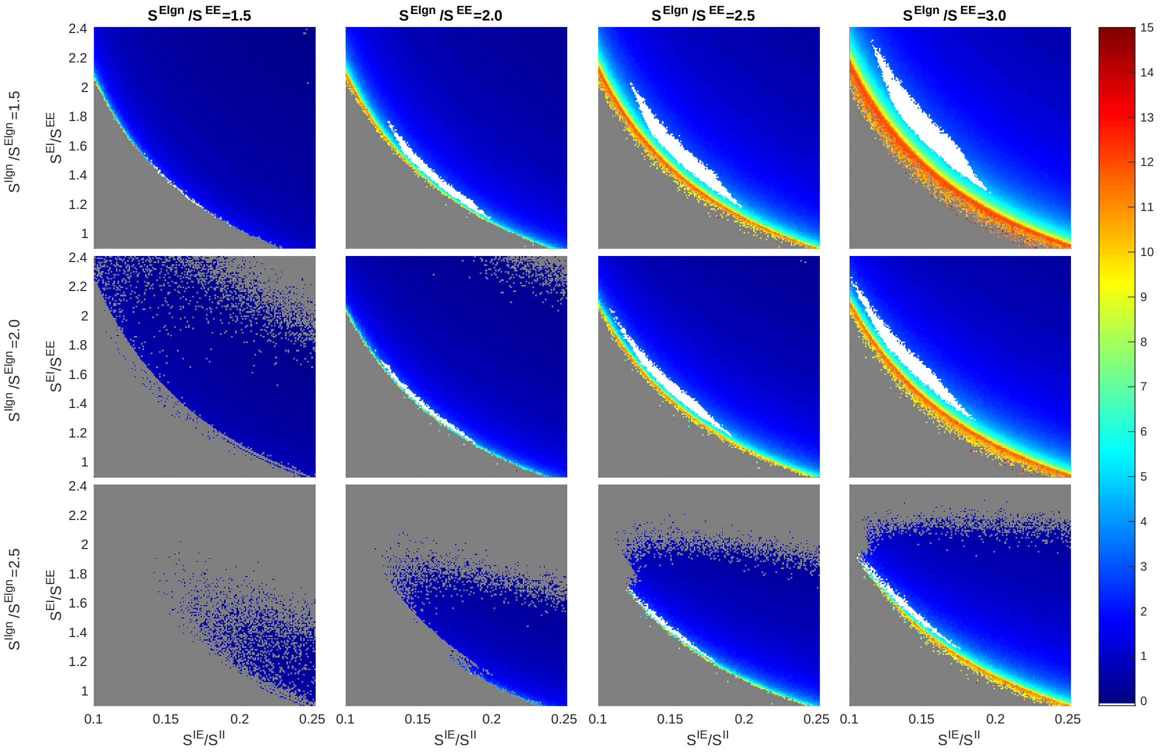

Figure F: A version of Fig 2 with $S^{EE} = 0.021$, $S^{II} = 0.12$, and $F^{IL6}/F^{EL6} = 3$.

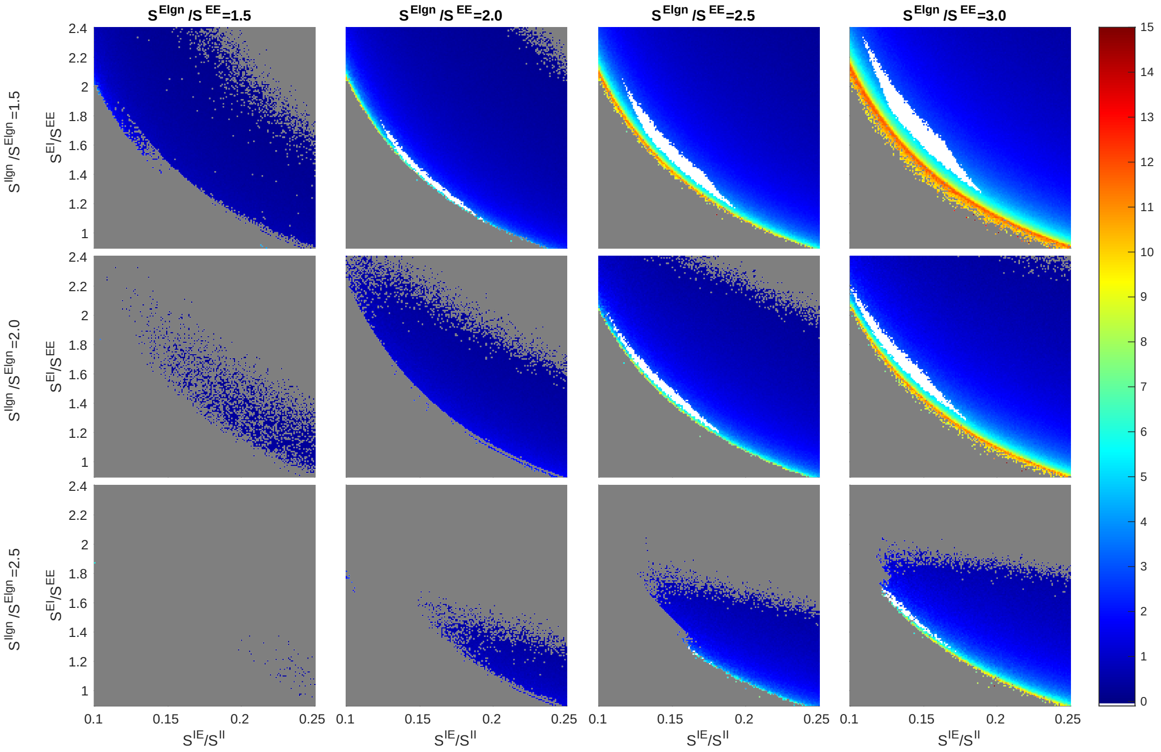

Figure G: A version of Fig 2 with $S^{EE} = 0.021$, $S^{II} = 0.12$, and $F^{IL6}/F^{EL6} = 4.5$.

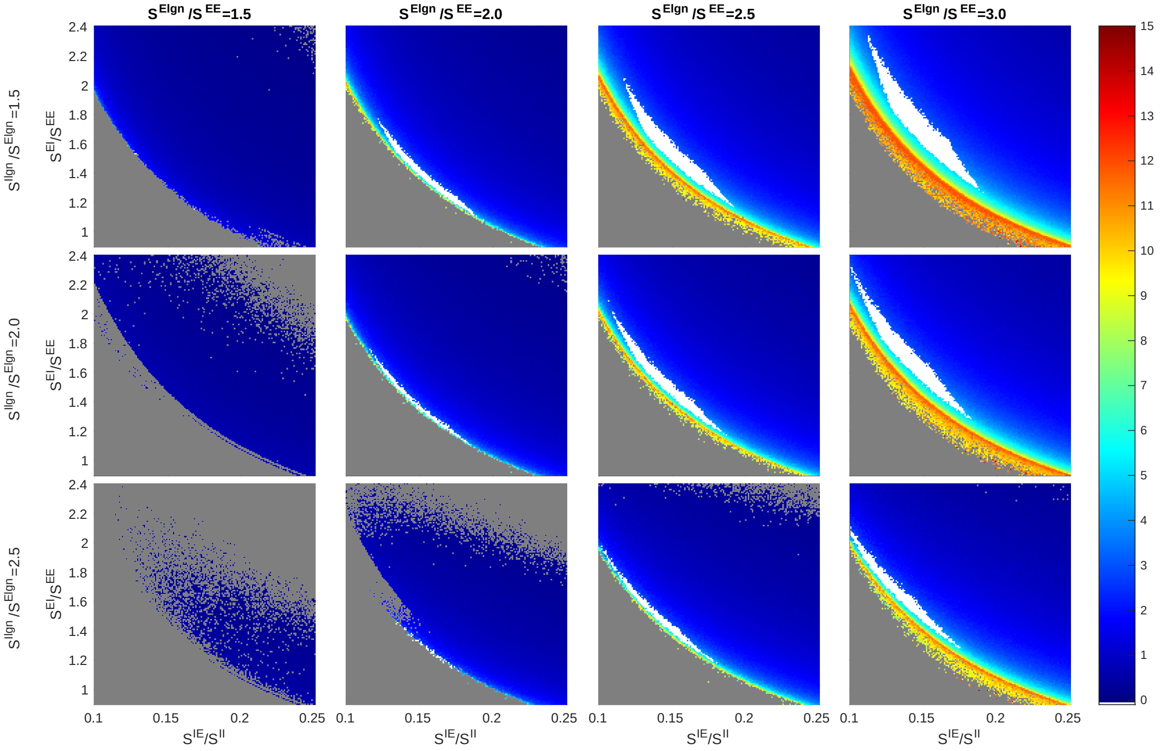

Figure H: A version of Fig 2 with $S^{EE} = 0.021$, $S^{II} = 0.16$, and $F^{IL6}/F^{EL6} = 3$.

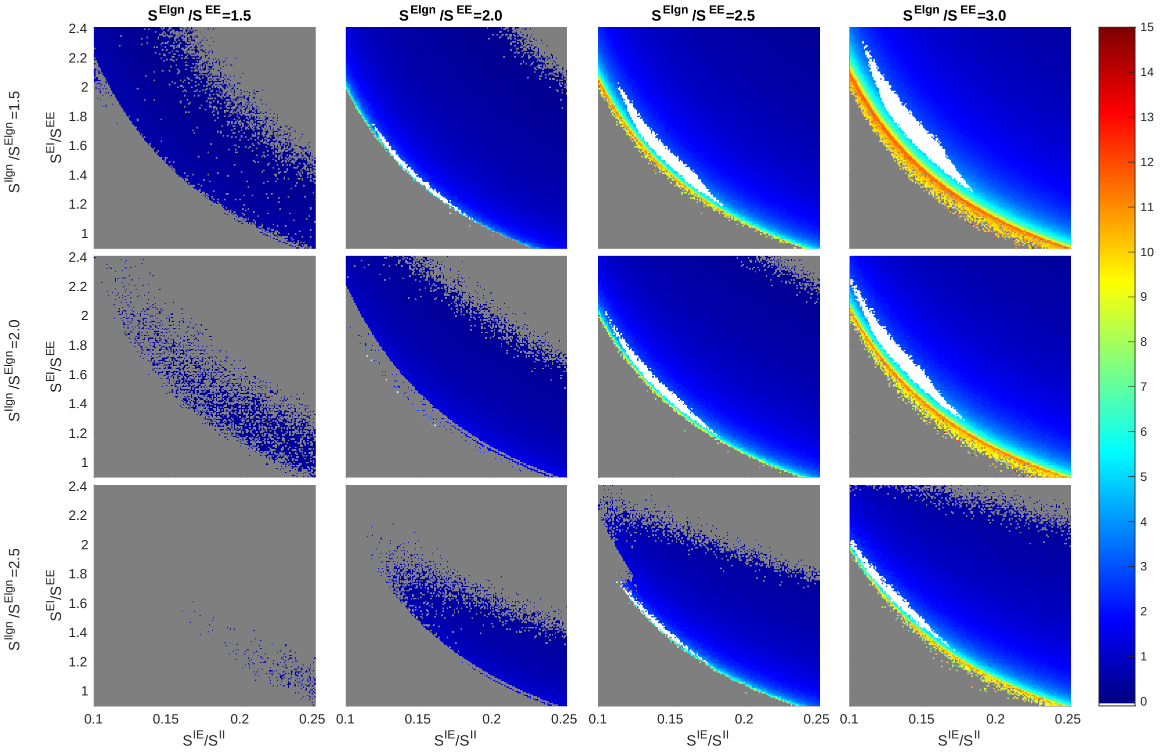

Figure I: A version of Fig 2 with $S^{EE} = 0.021$, $S^{II} = 0.16$, and $F^{IL6}/F^{EL6} = 4.5$.

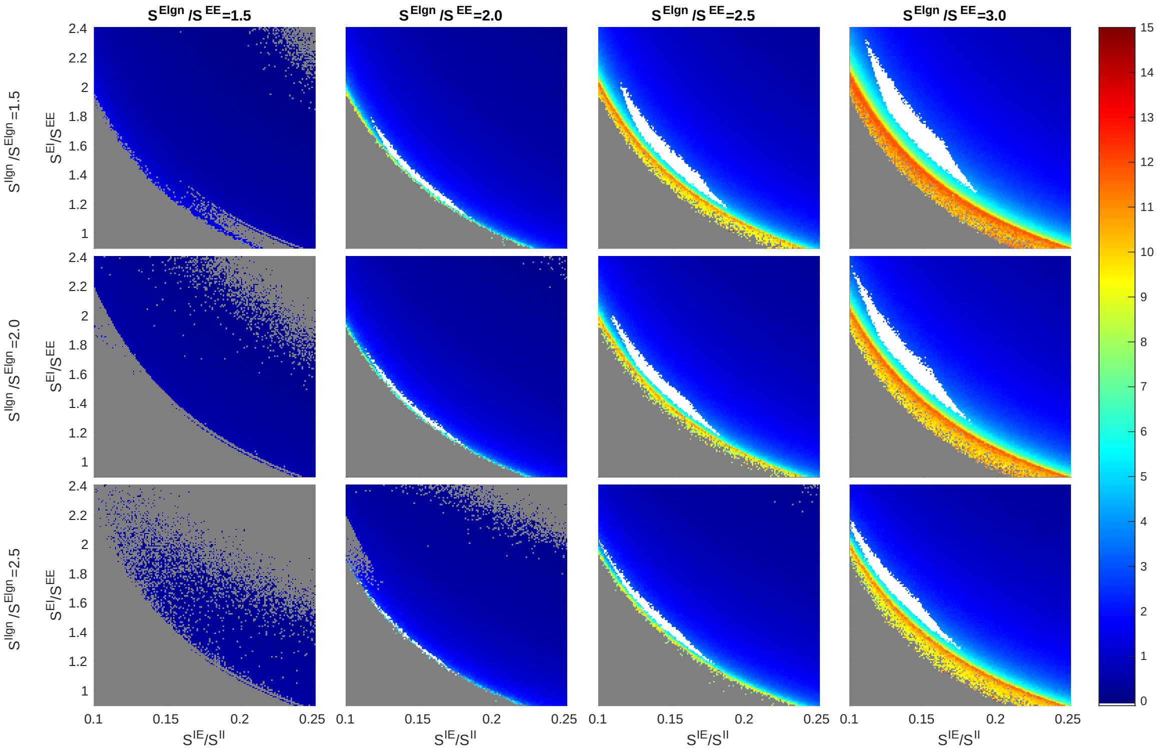

Figure J: A version of Fig 2 with $S^{EE} = 0.021$, $S^{II} = 0.20$, and $F^{IL6}/F^{EL6} = 3$.

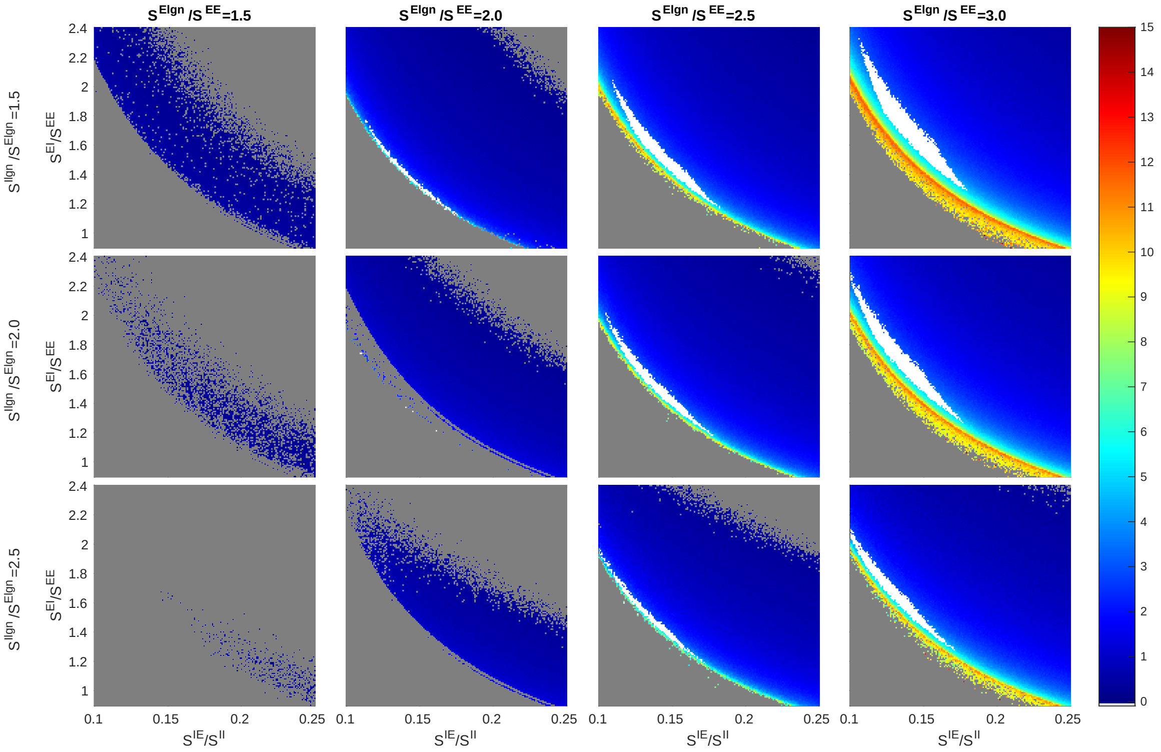

Figure K: A version of Fig 2 with $S^{EE} = 0.021$, $S^{II} = 0.20$, and $F^{IL6}/F^{EL6} = 4.5$.

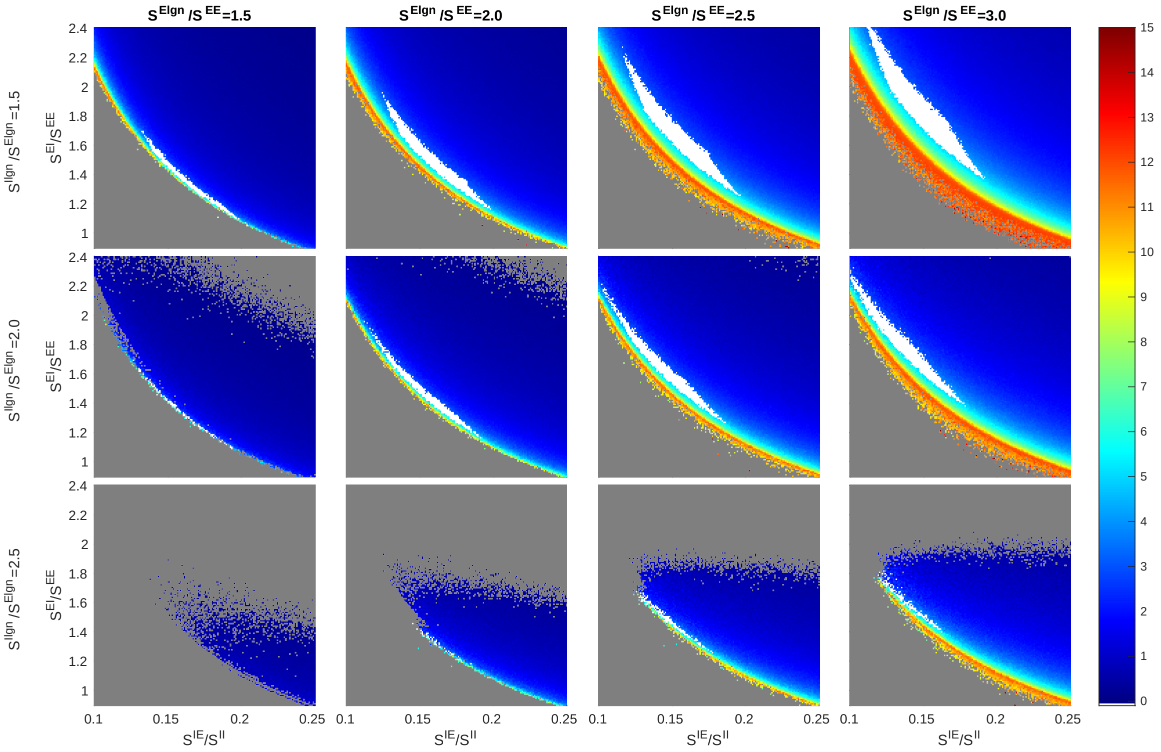

Figure L: A version of Fig 2 with $S^{EE} = 0.024$, $S^{II} = 0.12$, and $F^{IL6}/F^{EL6} = 3$.

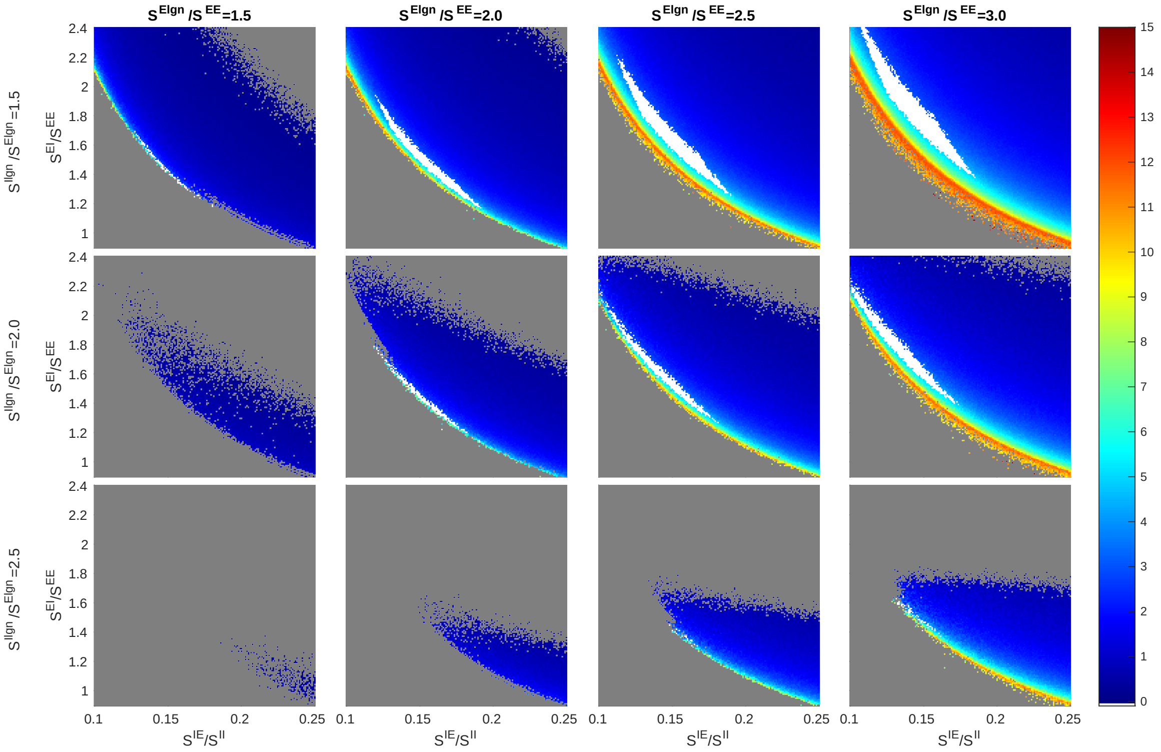

Figure M: A version of Fig 2 with $S^{EE} = 0.024$, $S^{II} = 0.12$, and $F^{IL6}/F^{EL6} = 4.5$.

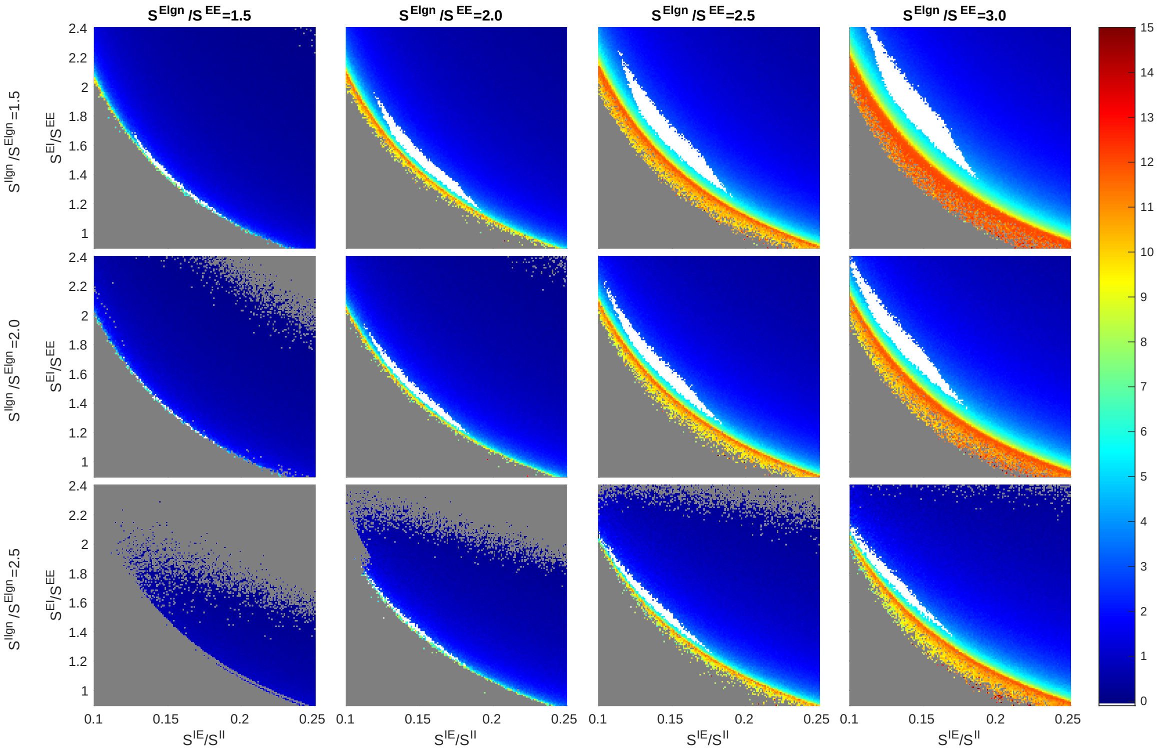

Figure N: A version of Fig 2 with $S^{EE} = 0.024$, $S^{II} = 0.16$, and $F^{IL6}/F^{EL6} = 3$.

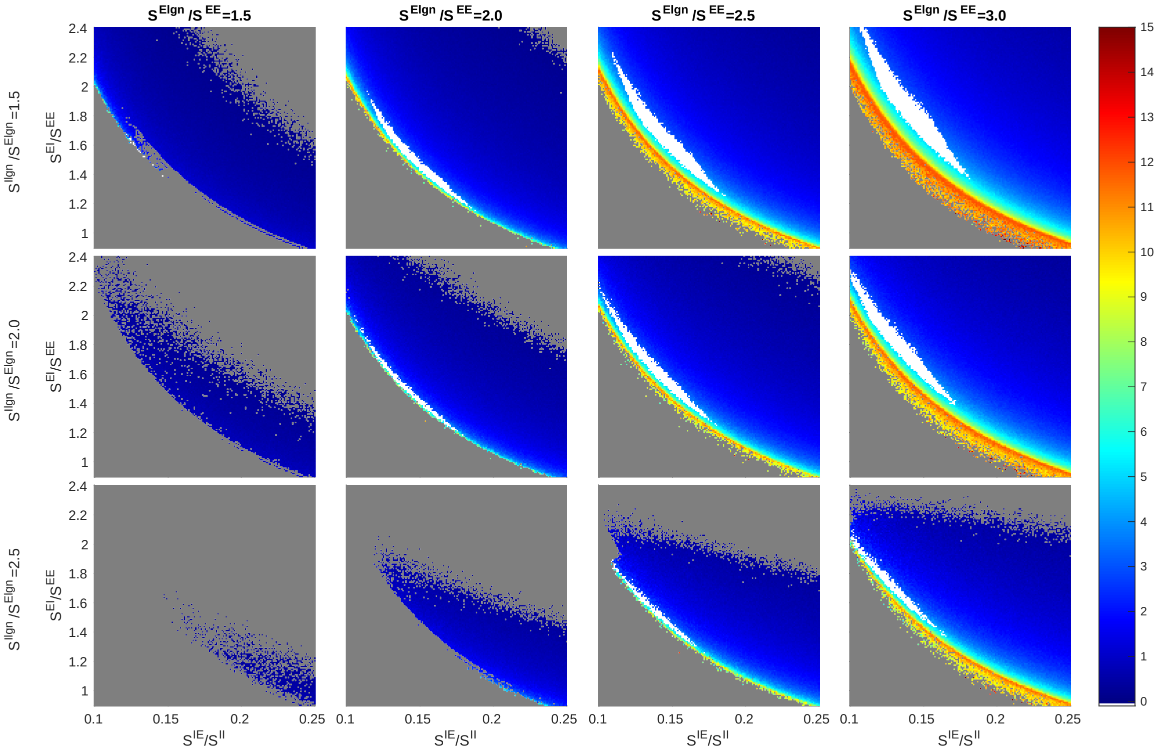

Figure O: A version of Fig 2 with $S^{EE} = 0.024$, $S^{II} = 0.16$, and $F^{IL6}/F^{EL6} = 4.5$.

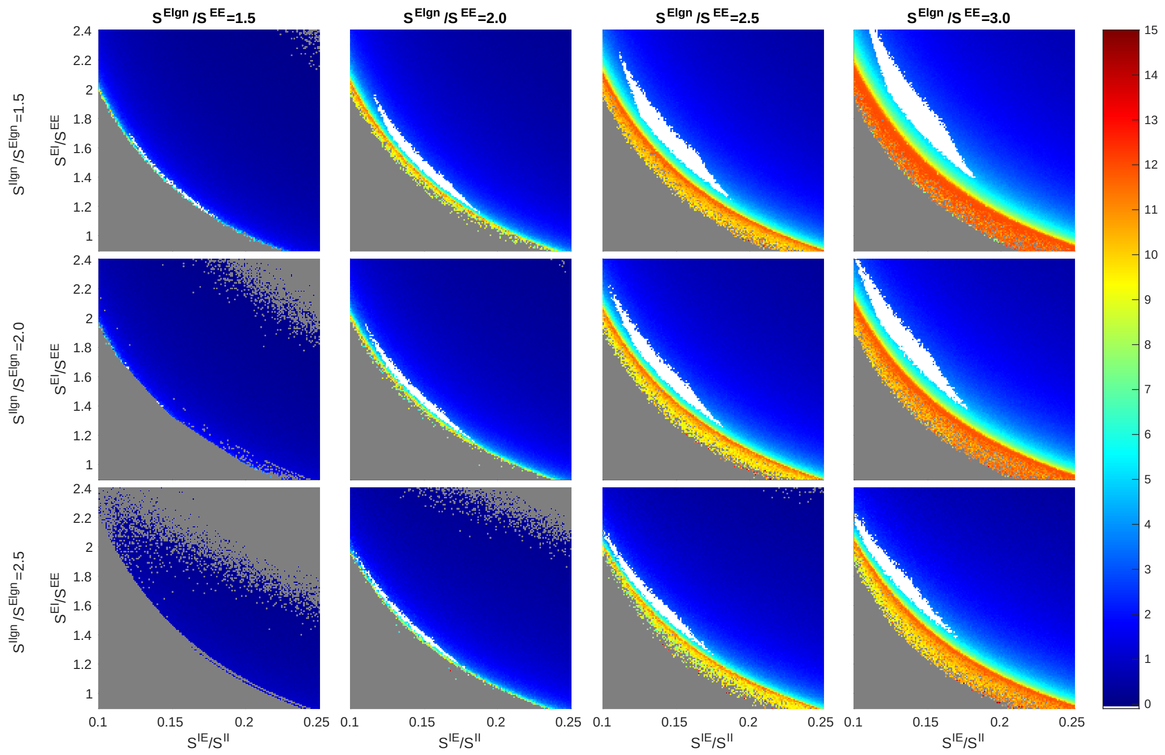

Figure P: A version of Fig 2 with $S^{EE} = 0.024$, $S^{II} = 0.20$, and $F^{IL6}/F^{EL6} = 3$.

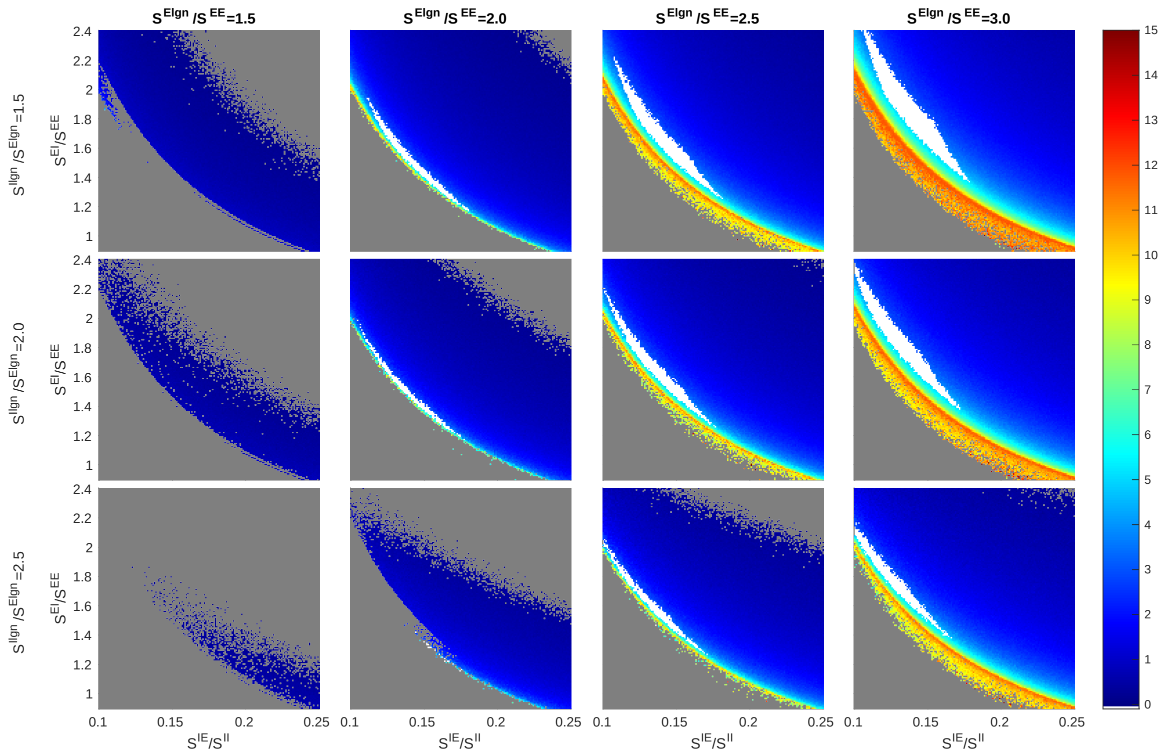

Figure Q: A version of Fig 2 with $S^{EE} = 0.024$, $S^{II} = 0.20$, and $F^{IL6}/F^{EL6} = 4.5$.

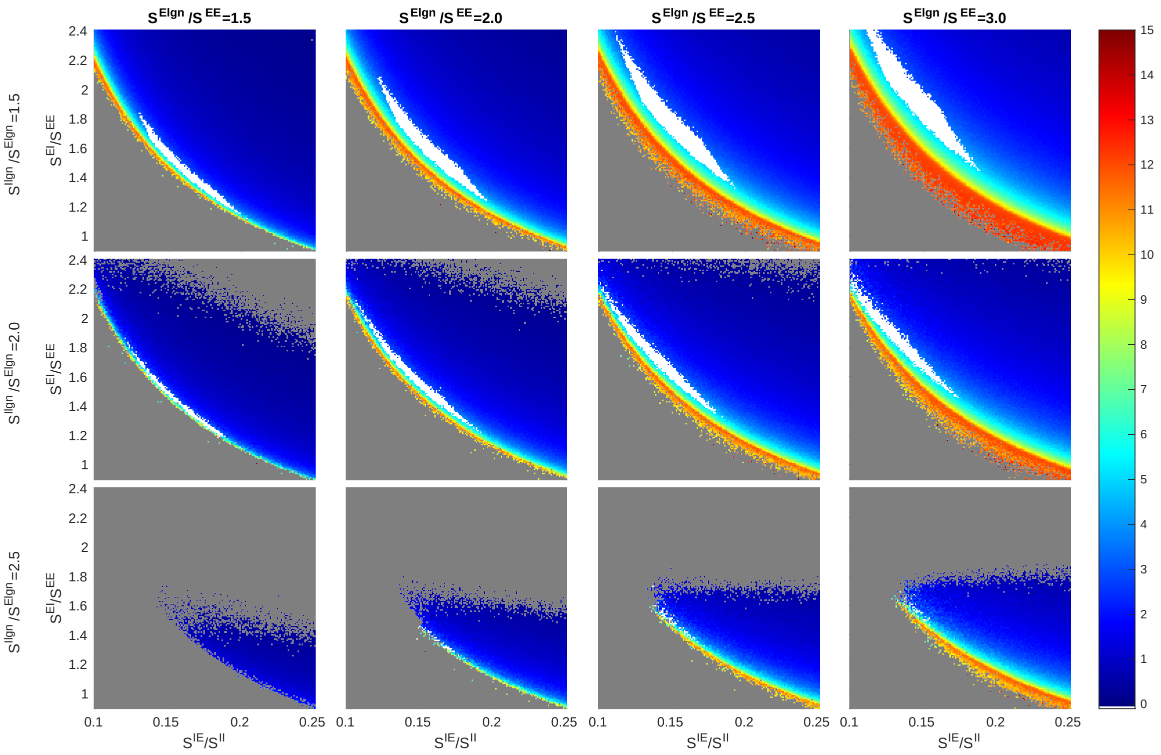

Figure R: A version of Fig 2 with $S^{EE} = 0.027$, $S^{II} = 0.12$, and $F^{IL6}/F^{EL6} = 3$.

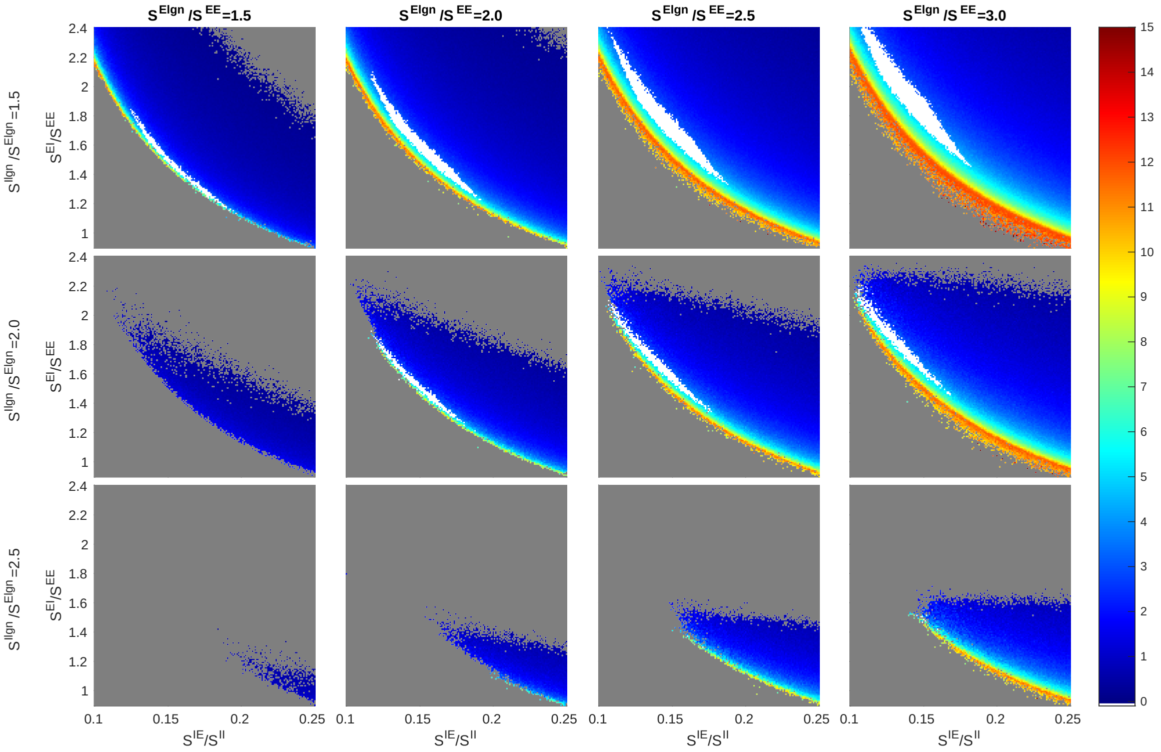

Figure S: A version of Fig 2 with $S^{EE} = 0.027$, $S^{II} = 0.12$, and $F^{IL6}/F^{EL6} = 4.5$.

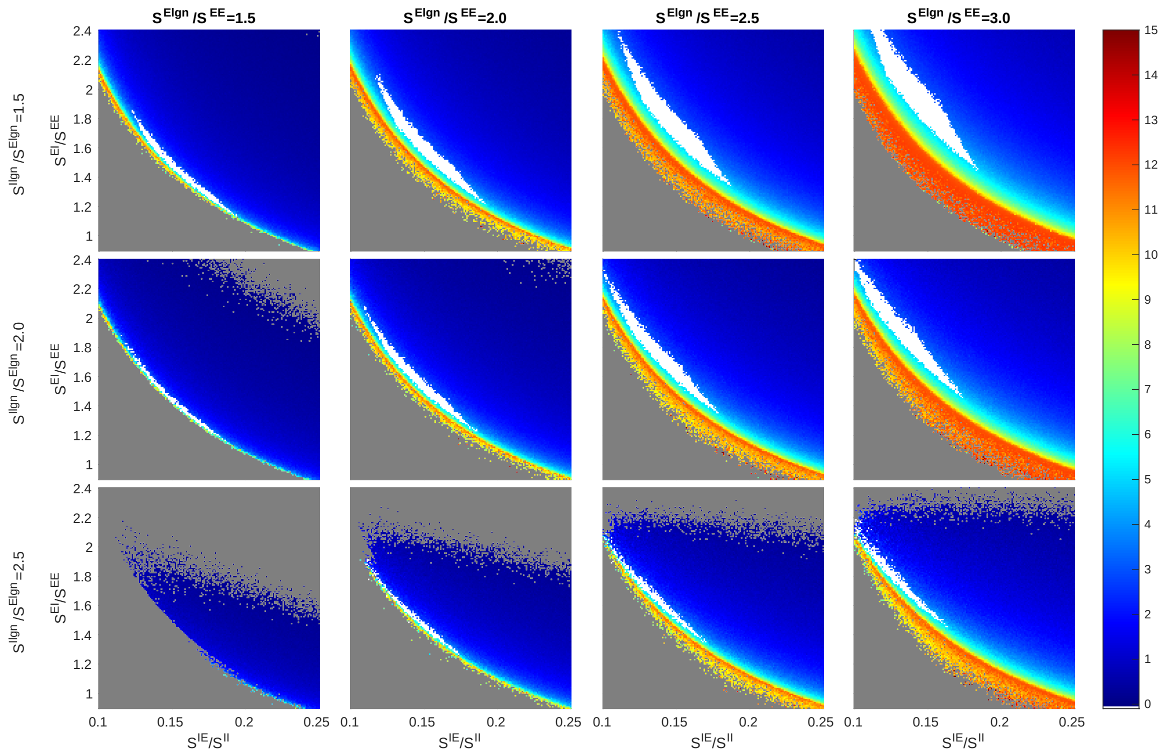

Figure T: A version of Fig 2 with $S^{EE} = 0.027$, $S^{II} = 0.16$, and $F^{IL6}/F^{EL6} = 3$.

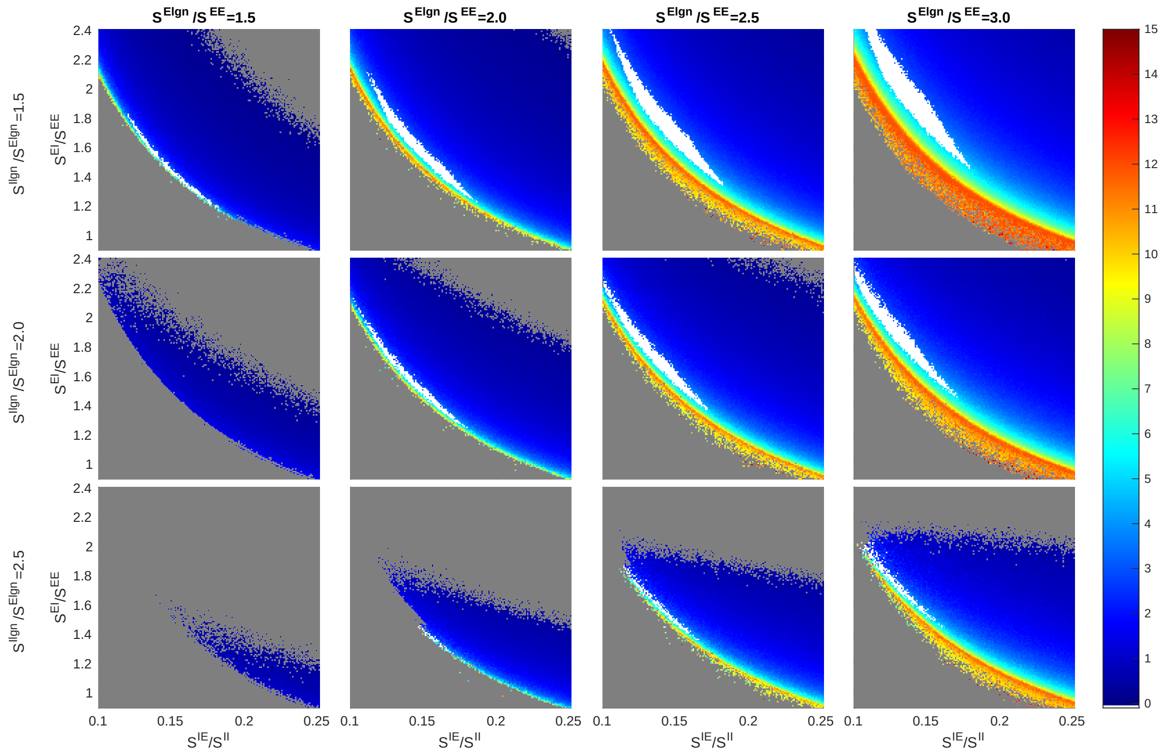

Figure U: A version of Fig 2 with $S^{EE} = 0.027$, $S^{II} = 0.16$, and $F^{IL6}/F^{EL6} = 4.5$.

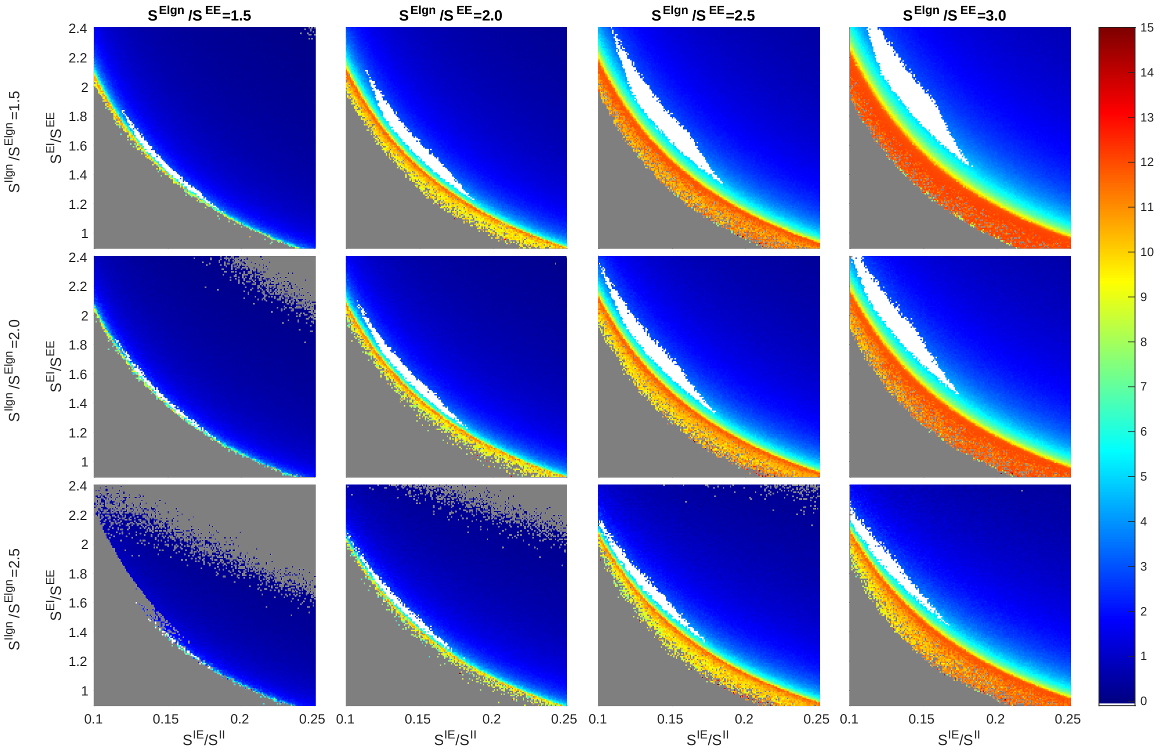

Figure V: A version of Fig 2 with $S^{EE} = 0.027$, $S^{II} = 0.20$, and $F^{IL6}/F^{EL6} = 3$.

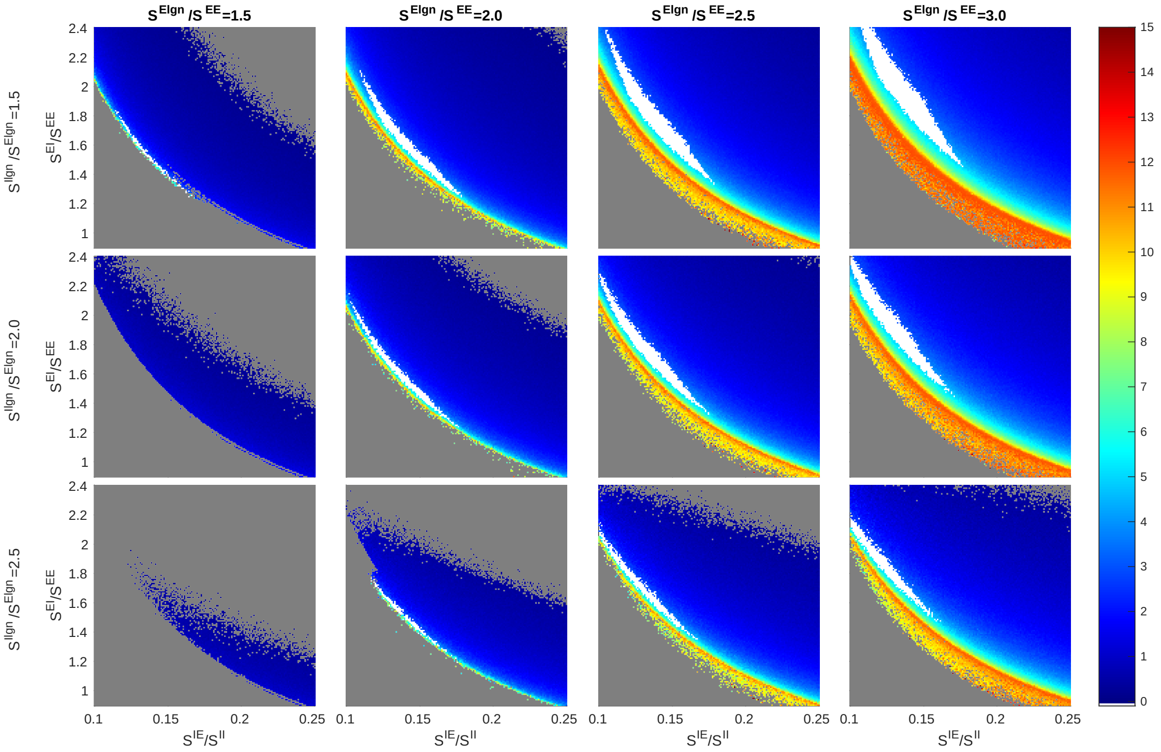

Figure W: A version of Fig 2 with $S^{EE} = 0.027$, $S^{II} = 0.20$, and $F^{IL6}/F^{EL6} = 4.5$.

References

[1] Chariker L, Shapley R, Young LS. Orientation selectivity from very sparse LGN inputs in a comprehensive model of macaque V1 cortex. Journal of Neuroscience. 2016;36(49):12368–12384.

[2] Koch C. Biophysics of computations; 1999.

[3] Chariker L, Shapley R, Young LS. Rhythm and synchrony in a cortical network model. Journal of Neuroscience. 2018;38(40):8621–8634.

[4] Chariker L, Young LS. Emergent spike patterns in neuronal populations. Journal of computational neuroscience. 2015;38(1):203–220.

[5] Levy RB, Reyes AD. Spatial profile of excitatory and inhibitory synaptic connectivity in mouse primary auditory cortex. Journal of Neuroscience. 2012;32(16):5609–5619.

[6] Stratford K, Tarczy-Hornoch K, Martin K, Bannister N, Jack J. Excitatory synaptic inputs to spiny stellate cells in cat visual cortex. Nature. 1996;382(6588):258–261.

[7] Beierlein M, Gibson JR, Connors BW. Two dynamically distinct inhibitory networks in layer 4 of the neocortex. Journal of neurophysiology. 2003;90(5):2987–3000.
